# The crystal structure of 1-(2-hydroxy-5-meth­oxy­phen­yl)ethanone 4,4-di­methyl­thio­semicarbazone

**DOI:** 10.1107/S2056989015018228

**Published:** 2015-10-03

**Authors:** Brian J. Anderson, Michael B. Freedman, Victoria A. Smolenski, Jerry P. Jasinski

**Affiliations:** a Department of Chemistry, Keene State College, 229 Main Street, Keene, NH 03435-2001, USA

**Keywords:** crystal structure, thio­semicarbazone, weak inter­molecular inter­actions, C—H⋯π inter­actions

## Abstract

The asymmetric unit of the title compound, C_12_H_17_N_3_O_2_S, contains two independent mol­ecules, *A* and *B*. Both mol­ecules are nearly planar with the dihedral angle between the mean planes of the thio­amide group and benzene ring being 7.5 (1)° in *A* and 4.3 (2)° in *B*. In each mol­ecule, the hy­droxy group participates in intra­molecular O—H⋯N hydrogen bonding, while the amino H atom is not involved in hydrogen bonding because of the steric hinderence caused by two neighboring methyl groups. In the crystal, the individual molecules are linked by weak C—H⋯O hydrogen bonds, forming *A*–*A* and *B*–*B* inversion dimers. The dimers are linked *via* C—H⋯π inter­actions which help stabilize the packing.

## Related literature   

For thio­semicarbazone ligands and metal complexes, see: Lobana *et al.* (2009 ▸, 2012 ▸). For biological and anti­tumor and anti­fungal activity of palladium complexes with thio­semicarbazone ligands, see: Chellan *et al.* (2010 ▸). For biological activity of a thio­semicarbazone ligand with a terminal dimethyl substitution, see: Kowol *et al.* (2009 ▸). For related structures, see: Anderson *et al.* (2012 ▸, 2013 ▸); Kovala-Demertzi *et al.* (2000 ▸).
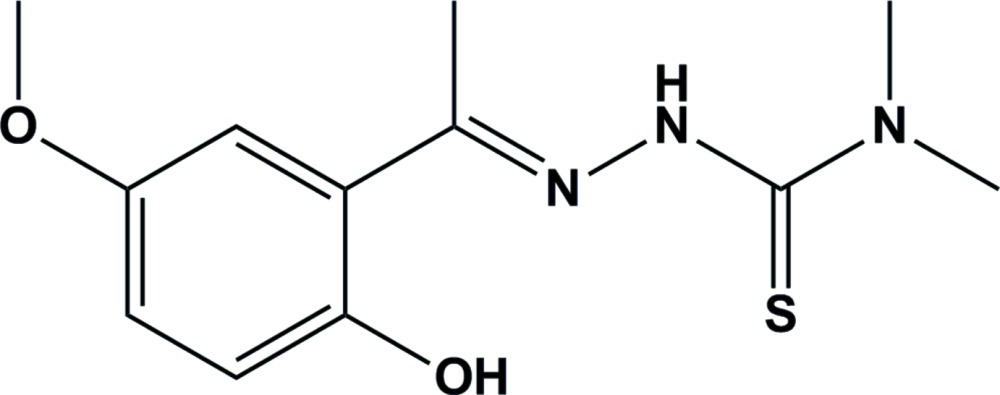


## Experimental   

### Crystal data  

C_12_H_17_N_3_O_2_S
*M* *_r_* = 267.34
Monoclinic 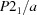

*a* = 15.7097 (12) Å
*b* = 7.8300 (5) Å
*c* = 21.2351 (19) Å
β = 92.635 (8)°
*V* = 2609.3 (3) Å^3^
*Z* = 8
Mo *K*α radiation
μ = 0.25 mm^−1^
*T* = 173 K
0.54 × 0.35 × 0.05 mm

### Data collection  

Agilent, Eos, Gemini diffractometer
Absorption correction: multi-scan (*CrysAlis PRO*; Agilent, 2014 ▸) *T*_min_ = 0.803, *T*_max_ = 1.000
33509 measured reflections
8982 independent reflections
6065 reflections with *I* > 2σ(*I*)
*R*_int_ = 0.081

### Refinement  

*R*[*F* ^2^ > 2σ(*F* ^2^)] = 0.078
*wR*(*F* ^2^) = 0.224
*S* = 1.06
8982 reflections
334 parameters
H-atom parameters constrained
Δρ_max_ = 1.08 e Å^−3^
Δρ_min_ = −0.48 e Å^−3^

### 

Data collection: *CrysAlis PRO* (Agilent, 2014 ▸); cell refinement: *CrysAlis PRO*; data reduction: *CrysAlis PRO*; program(s) used to solve structure: *SHELXS2014* (Sheldrick, 2008 ▸); program(s) used to refine structure: *SHELXL2014* (Sheldrick, 2015 ▸); molecular graphics: *OLEX2* (Dolomanov *et al.*, 2009 ▸); software used to prepare material for publication: *OLEX2*.

## Supplementary Material

Crystal structure: contains datablock(s) global, I. DOI: 10.1107/S2056989015018228/cv5496sup1.cif

Structure factors: contains datablock(s) I. DOI: 10.1107/S2056989015018228/cv5496Isup2.hkl

Supporting information file. DOI: 10.1107/S2056989015018228/cv5496Isup3.cml

**Fig. 1.**
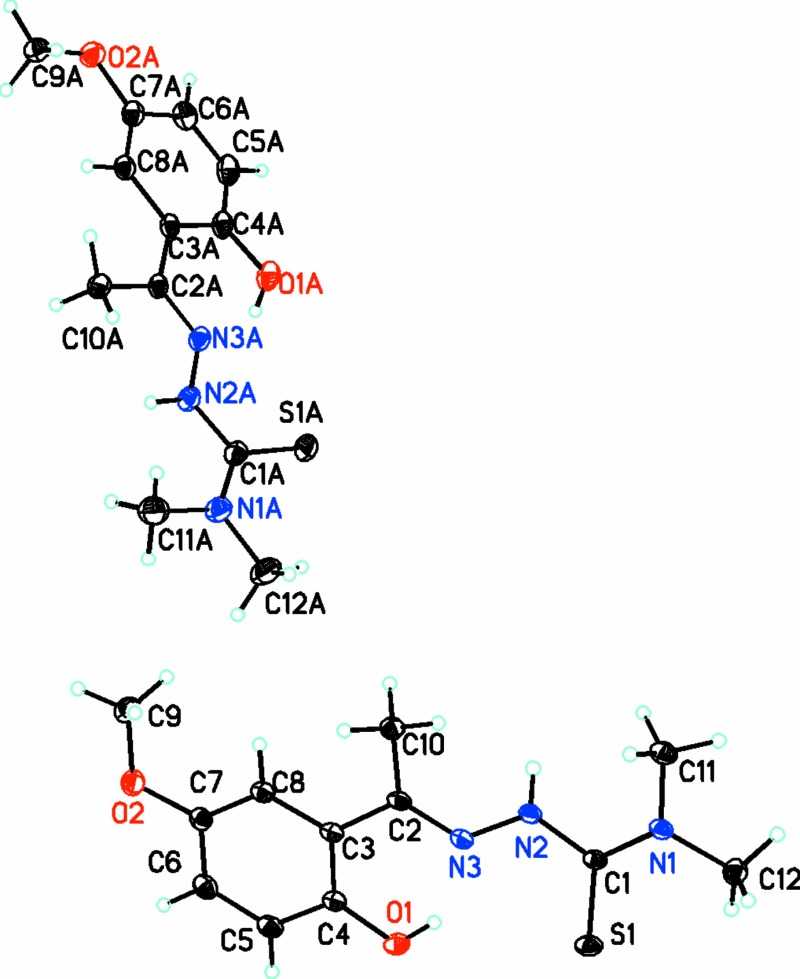
Two independent molecules of the title compound showing the atom-labelling scheme. Displacement ellipsoids are drawn at the 30% probability level.

**Fig. 2.**
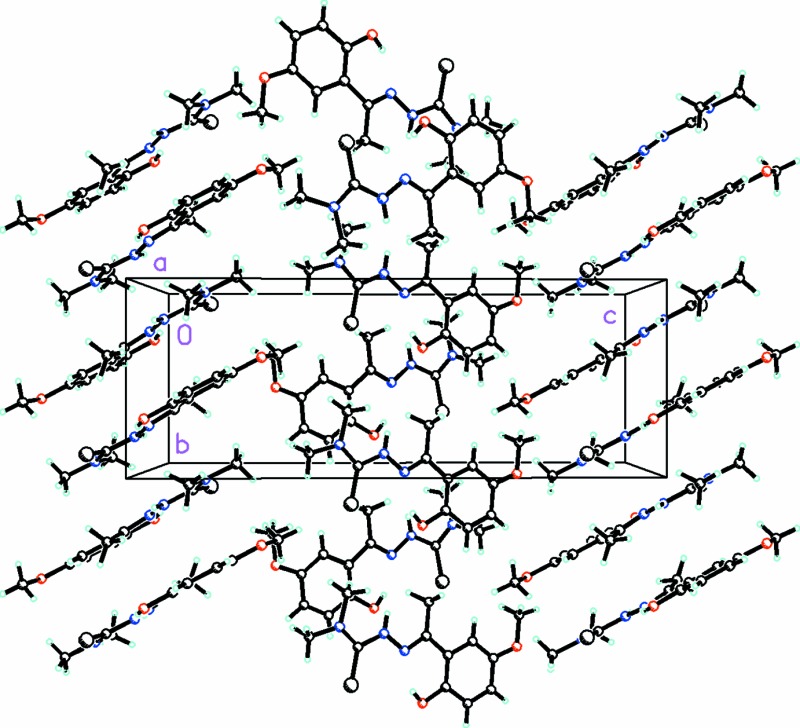
A portion of the crystal packing viewed approximately along the a axis.

. DOI: 10.1107/S2056989015018228/cv5496fig1.tif
Two independent mol­ecules of the title compound showing the atom-labelling scheme. Displacement ellipsoids are drawn at the 30% probability level.

a . DOI: 10.1107/S2056989015018228/cv5496fig2.tif
A portion of the crystal packing viewed approximately along the *a* axis.

CCDC reference: 1428535

Additional supporting information:  crystallographic information; 3D view; checkCIF report

## Figures and Tables

**Table 1:** Hydrogen-bond geometry (, ) *Cg*1 and *Cg*2 are the centroids of the C3C8 and C3*A*C8*A* rings, respectively.

*D*H*A*	*D*H	H*A*	*D* *A*	*D*H*A*
O1H1N3	0.84	1.84	2.563(2)	143
O1*A*H1*A*N3*A*	0.84	1.86	2.565(3)	141
C11H11*A*O1^i^	0.98	2.51	3.315(3)	139
C11*A*H11*E*O1*A* ^ii^	0.98	2.68	3.305(4)	122
C11*A*H11*E* *Cg*2^iii^	0.98	2.73	3.590(3)	147
C12H12*B* *Cg*1^i^	0.98	2.82	3.530(3)	130

Symmetry codes: (i) 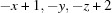; (ii) 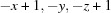; (iii) 
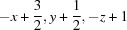
.

## supplementary crystallographic information

### S1. Comment

The asymmetric unit of the title compound, C_12_H_17_N_3_O_2_S, contains two independent molecules *A* and *B*, respectively (Fig. 1). Both molecules are nearly planar with the dihedral angle between the mean planes of the thioamide group and benzene ring being 7.5 (1)° in *A* and 4.3 (2)° in *B*. In each molecule, the hydroxy group participates in intramolecular O—H···N hydrogen bonding, while the amino H atom is not involved in hydrogen bonding because of the steric hinderence caused by two neighboring methyl groups. In the crystal, weak intermolecular C—H···O and C—H···π (Table 1) interactions are observed which help stabilize the packing (Fig. 2). No π—π stacking interactions are present.

### S2. Experimental

A 25 ml round bottom flask charged with 2.5 ml of H_2_O, 2.5 ml e thanol and 0.1499 g (1.26 mmol) of 4,4-dimethyl-3-thiosemicarbazide was dissolved in a water/ethanol mixture and heated. Once the mixture was completely dissolved, 0.2225 g (1.34 mmol) of 2'-hydroxy-5'-methoxyacetophenone was added, and the solution was refluxed for 18 h resulting in the formation of a yellow solid. After reflux, the slurry was allowed to cool to room temperature, transferred to a separatory funnel and water (15 ml) and dichloromethane (15 ml) was added. The organic layer was separated and the aqueous layer was extracted with an additional 15 ml of DCM. The organic layers were then combined and washed with brine (20 ml), and then dried with magnesium sulfate. The solvent was removed by rotary evaporation. The resulting solid was crystallized from acetonitrile to give 67 mg (18% yield) of yellow crystals. The crystals were observed to decompose above 460 K.

### S3. Refinement

Crystal data, data collection and structure refinement details are summarized in Table 1. A l l H atoms were located in difference maps. The C–H and N–H atoms were treated as riding atoms in geometrically idealized positions with C—H, N—H distances of 0.95 Å, 0.88 Å and refined with *U*_iso_(H) = 1.2*U*_eq_(C, N). The CH_3_ and O–H atoms were also treated as riding atoms in geometrically idealized positions with the CH_3_, O—H distances of 0.98 Å, 0.84 Å and refined with *U*_iso_(H) = 1.5*U*_eq_(C, O).

### Crystal data

**Table:**

C_12_H_17_N_3_O_2_S	*F*(000) = 1136
*M**_r_* = 267.34	*D*_x_ = 1.361 Mg m^−^^3^
Monoclinic, *P*2_1_/*a*	Mo *K*α radiation, λ = 0.71073 Å
*a* = 15.7097 (12) Å	Cell parameters from 7195 reflections
*b* = 7.8300 (5) Å	θ = 3.0–32.8°
*c* = 21.2351 (19) Å	µ = 0.25 mm^−^^1^
β = 92.635 (8)°	*T* = 173 K
*V* = 2609.3 (3) Å^3^	Prism, colourless
*Z* = 8	0.54 × 0.35 × 0.05 mm

### Data collection

**Table:**

Agilent, Eos, Gemini diffractometer	8982 independent reflections
Radiation source: Enhance (Mo) X-ray Source	6065 reflections with *I* > 2σ(*I*)
Graphite monochromator	*R*_int_ = 0.081
Detector resolution: 16.0416 pixels mm^-1^	θ_max_ = 32.9°, θ_min_ = 3.0°
ω scans	*h* = −23→23
Absorption correction: multi-scan (*CrysAlis PRO*; Agilent, 2014)	*k* = −11→11
*T*_min_ = 0.803, *T*_max_ = 1.000	*l* = −30→28
33509 measured reflections	

### Refinement

**Table:**

Refinement on *F*^2^	Primary atom site location: structure-invariant direct methods
Least-squares matrix: full	Hydrogen site location: inferred from neighbouring sites
*R*[*F*^2^ > 2σ(*F*^2^)] = 0.078	H-atom parameters constrained
*wR*(*F*^2^) = 0.224	*w* = 1/[σ^2^(*F*_o_^2^) + (0.1106*P*)^2^ + 0.7177*P*] where *P* = (*F*_o_^2^ + 2*F*_c_^2^)/3
*S* = 1.06	(Δ/σ)_max_ = 0.001
8982 reflections	Δρ_max_ = 1.08 e Å^−^^3^
334 parameters	Δρ_min_ = −0.48 e Å^−^^3^
0 restraints	

### Special details

**Table:**

Experimental. Absorption correction: *CrysAlis PRO* (Agilent, 2014). Empirical absorption correction using spherical harmonics, implemented in SCALE3 ABSPACK scaling algorithm.
Geometry. All e.s.d.'s (except the e.s.d. in the dihedral angle between two l.s. planes) are estimated using the full covariance matrix. The cell e.s.d.'s are taken into account individually in the estimation of e.s.d.'s in distances, angles and torsion angles; correlations between e.s.d.'s in cell parameters are only used when they are defined by crystal symmetry. An approximate (isotropic) treatment of cell e.s.d.'s is used for estimating e.s.d.'s involving l.s. planes.

### Fractional atomic coordinates and isotropic or equivalent isotropic displacement parameters (Å^2^)

**Table:**

	*x*	*y*	*z*	*U*_iso_*/*U*_eq_	
S1	0.44809 (3)	0.09151 (7)	1.11976 (3)	0.03013 (15)	
O1	0.32782 (10)	0.2857 (2)	0.99601 (7)	0.0293 (3)	
H1	0.3742	0.2469	1.0112	0.044*	
O2	0.35386 (11)	0.6134 (2)	0.76763 (7)	0.0336 (4)	
N1	0.61624 (11)	0.0389 (2)	1.11969 (8)	0.0248 (3)	
N2	0.55831 (11)	0.1799 (2)	1.03407 (8)	0.0246 (3)	
H2	0.6099	0.1881	1.0199	0.030*	
N3	0.48981 (11)	0.2446 (2)	1.00018 (8)	0.0233 (3)	
C1	0.54532 (13)	0.1017 (2)	1.09064 (9)	0.0215 (4)	
C2	0.50099 (12)	0.3329 (2)	0.94986 (9)	0.0220 (4)	
C3	0.42248 (12)	0.3932 (2)	0.91666 (9)	0.0211 (4)	
C4	0.34139 (13)	0.3677 (3)	0.94099 (9)	0.0236 (4)	
C5	0.26923 (14)	0.4287 (3)	0.90750 (11)	0.0295 (4)	
H5	0.2148	0.4145	0.9246	0.035*	
C6	0.27513 (14)	0.5088 (3)	0.85053 (11)	0.0299 (4)	
H6	0.2251	0.5482	0.8282	0.036*	
C7	0.35447 (13)	0.5322 (3)	0.82530 (10)	0.0258 (4)	
C8	0.42704 (13)	0.4770 (3)	0.85839 (9)	0.0247 (4)	
H8	0.4811	0.4960	0.8414	0.030*	
C9	0.43341 (17)	0.6297 (4)	0.73913 (11)	0.0405 (6)	
H9A	0.4248	0.6843	0.6978	0.061*	
H9B	0.4585	0.5162	0.7339	0.061*	
H9C	0.4719	0.6997	0.7660	0.061*	
C10	0.58601 (13)	0.3769 (3)	0.92477 (10)	0.0267 (4)	
H10A	0.6306	0.3603	0.9580	0.040*	
H10B	0.5858	0.4963	0.9110	0.040*	
H10C	0.5971	0.3026	0.8889	0.040*	
C11	0.70021 (13)	0.0579 (3)	1.09373 (10)	0.0292 (4)	
H11A	0.7003	0.0029	1.0523	0.044*	
H11B	0.7432	0.0041	1.1221	0.044*	
H11C	0.7134	0.1795	1.0894	0.044*	
C12	0.61403 (15)	−0.0437 (3)	1.18084 (10)	0.0302 (4)	
H12A	0.6294	0.0392	1.2140	0.045*	
H12B	0.6547	−0.1386	1.1828	0.045*	
H12C	0.5565	−0.0873	1.1869	0.045*	
S1A	0.56414 (5)	−0.19363 (10)	0.59613 (3)	0.0475 (2)	
O1A	0.66714 (13)	−0.3267 (2)	0.44906 (9)	0.0443 (5)	
H1A	0.6392	−0.2552	0.4692	0.066*	
O2A	0.82584 (12)	−0.0500 (2)	0.24624 (8)	0.0411 (4)	
N1A	0.53156 (14)	0.1371 (3)	0.61861 (10)	0.0413 (5)	
N2A	0.59826 (13)	0.0826 (3)	0.52876 (9)	0.0357 (4)	
H2A	0.5970	0.1935	0.5220	0.043*	
N3A	0.63480 (12)	−0.0228 (3)	0.48627 (9)	0.0319 (4)	
C1A	0.56361 (15)	0.0174 (4)	0.58155 (11)	0.0355 (5)	
C2A	0.67559 (14)	0.0493 (3)	0.44166 (10)	0.0287 (4)	
C3A	0.71116 (14)	−0.0682 (3)	0.39541 (10)	0.0280 (4)	
C4A	0.70414 (16)	−0.2470 (3)	0.40023 (11)	0.0349 (5)	
C5A	0.73558 (19)	−0.3507 (3)	0.35358 (12)	0.0421 (6)	
H5A	0.7299	−0.4711	0.3568	0.050*	
C6A	0.77470 (18)	−0.2826 (3)	0.30294 (12)	0.0406 (6)	
H6A	0.7957	−0.3555	0.2714	0.049*	
C7A	0.78354 (16)	−0.1063 (3)	0.29783 (11)	0.0334 (5)	
C8A	0.75226 (15)	−0.0007 (3)	0.34303 (11)	0.0311 (4)	
H8A	0.7584	0.1195	0.3390	0.037*	
C9A	0.83626 (17)	0.1293 (4)	0.24074 (12)	0.0396 (5)	
H9AA	0.8701	0.1720	0.2773	0.059*	
H9AB	0.7802	0.1845	0.2389	0.059*	
H9AC	0.8656	0.1550	0.2021	0.059*	
C10A	0.68571 (17)	0.2386 (3)	0.43561 (12)	0.0357 (5)	
H10D	0.6873	0.2908	0.4776	0.054*	
H10E	0.6375	0.2851	0.4101	0.054*	
H10F	0.7389	0.2637	0.4151	0.054*	
C11A	0.5344 (2)	0.3188 (4)	0.60277 (14)	0.0511 (7)	
H11D	0.5078	0.3852	0.6357	0.077*	
H11E	0.5035	0.3382	0.5623	0.077*	
H11F	0.5939	0.3547	0.5998	0.077*	
C12A	0.4973 (2)	0.0922 (5)	0.67918 (14)	0.0571 (8)	
H12D	0.5362	0.1325	0.7134	0.086*	
H12E	0.4912	−0.0321	0.6820	0.086*	
H12F	0.4414	0.1462	0.6828	0.086*	

### Atomic displacement parameters (Å^2^)

**Table:**

	*U*^11^	*U*^22^	*U*^33^	*U*^12^	*U*^13^	*U*^23^
S1	0.0221 (3)	0.0347 (3)	0.0344 (3)	−0.0020 (2)	0.01022 (19)	0.00048 (19)
O1	0.0209 (7)	0.0346 (9)	0.0330 (8)	−0.0050 (6)	0.0082 (6)	0.0030 (6)
O2	0.0299 (8)	0.0352 (9)	0.0358 (8)	−0.0002 (7)	0.0011 (6)	0.0072 (6)
N1	0.0191 (8)	0.0243 (8)	0.0313 (9)	0.0007 (6)	0.0058 (6)	0.0012 (6)
N2	0.0204 (8)	0.0245 (8)	0.0295 (8)	0.0024 (6)	0.0079 (6)	0.0015 (6)
N3	0.0206 (8)	0.0212 (8)	0.0284 (8)	0.0020 (6)	0.0043 (6)	−0.0025 (6)
C1	0.0209 (9)	0.0178 (8)	0.0263 (9)	−0.0008 (6)	0.0050 (7)	−0.0035 (6)
C2	0.0208 (9)	0.0179 (8)	0.0277 (9)	−0.0001 (7)	0.0067 (7)	−0.0049 (6)
C3	0.0178 (8)	0.0168 (8)	0.0290 (9)	−0.0024 (6)	0.0047 (7)	−0.0040 (6)
C4	0.0214 (9)	0.0196 (8)	0.0303 (10)	−0.0028 (7)	0.0053 (7)	−0.0035 (7)
C5	0.0184 (9)	0.0295 (10)	0.0410 (12)	−0.0021 (8)	0.0067 (8)	−0.0009 (8)
C6	0.0220 (10)	0.0278 (10)	0.0396 (12)	−0.0004 (8)	−0.0011 (8)	−0.0004 (8)
C7	0.0261 (10)	0.0195 (9)	0.0317 (10)	−0.0008 (7)	0.0020 (7)	−0.0004 (7)
C8	0.0219 (9)	0.0212 (9)	0.0314 (10)	−0.0013 (7)	0.0049 (7)	−0.0028 (7)
C9	0.0376 (13)	0.0515 (16)	0.0330 (12)	0.0026 (11)	0.0066 (9)	0.0089 (10)
C10	0.0200 (9)	0.0272 (10)	0.0333 (10)	−0.0021 (7)	0.0050 (7)	0.0037 (7)
C11	0.0197 (9)	0.0314 (11)	0.0370 (11)	−0.0006 (8)	0.0051 (8)	−0.0012 (8)
C12	0.0307 (11)	0.0297 (11)	0.0300 (10)	−0.0010 (9)	0.0009 (8)	0.0034 (8)
S1A	0.0435 (4)	0.0464 (4)	0.0531 (4)	−0.0001 (3)	0.0089 (3)	0.0232 (3)
O1A	0.0528 (12)	0.0284 (9)	0.0515 (11)	−0.0099 (8)	0.0010 (8)	0.0119 (7)
O2A	0.0469 (11)	0.0383 (10)	0.0388 (9)	0.0026 (8)	0.0079 (7)	0.0008 (7)
N1A	0.0350 (12)	0.0492 (13)	0.0402 (11)	−0.0032 (10)	0.0064 (9)	0.0068 (9)
N2A	0.0370 (11)	0.0329 (10)	0.0375 (11)	−0.0036 (8)	0.0050 (8)	0.0086 (7)
N3A	0.0310 (10)	0.0310 (10)	0.0336 (10)	−0.0049 (8)	0.0004 (7)	0.0070 (7)
C1A	0.0237 (11)	0.0454 (14)	0.0373 (12)	−0.0057 (9)	0.0001 (8)	0.0117 (9)
C2A	0.0254 (10)	0.0248 (10)	0.0352 (11)	−0.0042 (8)	−0.0036 (8)	0.0084 (8)
C3A	0.0257 (10)	0.0233 (9)	0.0343 (11)	−0.0039 (8)	−0.0053 (8)	0.0055 (7)
C4A	0.0356 (12)	0.0273 (11)	0.0408 (12)	−0.0071 (9)	−0.0075 (9)	0.0079 (9)
C5A	0.0529 (16)	0.0243 (11)	0.0480 (14)	−0.0046 (10)	−0.0079 (11)	0.0022 (9)
C6A	0.0466 (15)	0.0311 (12)	0.0432 (13)	0.0005 (10)	−0.0060 (11)	−0.0047 (9)
C7A	0.0342 (12)	0.0317 (12)	0.0338 (11)	−0.0026 (9)	−0.0048 (9)	0.0020 (8)
C8A	0.0297 (11)	0.0268 (10)	0.0364 (11)	−0.0018 (8)	−0.0026 (8)	0.0046 (8)
C9A	0.0350 (13)	0.0426 (14)	0.0416 (13)	−0.0040 (11)	0.0070 (10)	0.0037 (10)
C10A	0.0385 (13)	0.0264 (11)	0.0428 (13)	−0.0029 (9)	0.0083 (10)	0.0061 (9)
C11A	0.0501 (18)	0.0504 (18)	0.0536 (17)	−0.0002 (13)	0.0090 (13)	−0.0007 (12)
C12A	0.0508 (18)	0.079 (2)	0.0424 (16)	−0.0007 (16)	0.0133 (13)	0.0123 (14)

### Geometric parameters (Å, º)

**Table:**

S1—C1	1.676 (2)	S1A—C1A	1.681 (3)
O1—H1	0.8400	O1A—H1A	0.8400
O1—C4	1.359 (2)	O1A—C4A	1.363 (3)
O2—C7	1.380 (3)	O2A—C7A	1.379 (3)
O2—C9	1.419 (3)	O2A—C9A	1.419 (3)
N1—C1	1.342 (3)	N1A—C1A	1.337 (4)
N1—C11	1.461 (3)	N1A—C11A	1.463 (4)
N1—C12	1.452 (3)	N1A—C12A	1.460 (3)
N2—H2	0.8800	N2A—H2A	0.8800
N2—N3	1.364 (2)	N2A—N3A	1.368 (3)
N2—C1	1.372 (2)	N2A—C1A	1.367 (3)
N3—C2	1.292 (3)	N3A—C2A	1.297 (3)
C2—C3	1.470 (3)	C2A—C3A	1.474 (3)
C2—C10	1.500 (3)	C2A—C10A	1.496 (3)
C3—C4	1.411 (3)	C3A—C4A	1.408 (3)
C3—C8	1.405 (3)	C3A—C8A	1.414 (3)
C4—C5	1.395 (3)	C4A—C5A	1.389 (4)
C5—H5	0.9500	C5A—H5A	0.9500
C5—C6	1.369 (3)	C5A—C6A	1.371 (4)
C6—H6	0.9500	C6A—H6A	0.9500
C6—C7	1.391 (3)	C6A—C7A	1.392 (3)
C7—C8	1.381 (3)	C7A—C8A	1.375 (3)
C8—H8	0.9500	C8A—H8A	0.9500
C9—H9A	0.9800	C9A—H9AA	0.9800
C9—H9B	0.9800	C9A—H9AB	0.9800
C9—H9C	0.9800	C9A—H9AC	0.9800
C10—H10A	0.9800	C10A—H10D	0.9800
C10—H10B	0.9800	C10A—H10E	0.9800
C10—H10C	0.9800	C10A—H10F	0.9800
C11—H11A	0.9800	C11A—H11D	0.9800
C11—H11B	0.9800	C11A—H11E	0.9800
C11—H11C	0.9800	C11A—H11F	0.9800
C12—H12A	0.9800	C12A—H12D	0.9800
C12—H12B	0.9800	C12A—H12E	0.9800
C12—H12C	0.9800	C12A—H12F	0.9800
			
C4—O1—H1	109.5	C4A—O1A—H1A	109.5
C7—O2—C9	116.72 (17)	C7A—O2A—C9A	116.31 (19)
C1—N1—C11	122.29 (17)	C1A—N1A—C11A	122.0 (2)
C1—N1—C12	121.38 (17)	C1A—N1A—C12A	121.0 (3)
C12—N1—C11	116.23 (17)	C12A—N1A—C11A	116.9 (3)
N3—N2—H2	120.6	N3A—N2A—H2A	119.6
N3—N2—C1	118.84 (17)	C1A—N2A—H2A	119.6
C1—N2—H2	120.6	C1A—N2A—N3A	120.7 (2)
C2—N3—N2	120.09 (17)	C2A—N3A—N2A	117.1 (2)
N1—C1—S1	124.37 (15)	N1A—C1A—S1A	125.40 (19)
N1—C1—N2	114.36 (17)	N1A—C1A—N2A	113.3 (2)
N2—C1—S1	121.27 (15)	N2A—C1A—S1A	121.3 (2)
N3—C2—C3	115.20 (17)	N3A—C2A—C3A	115.5 (2)
N3—C2—C10	125.01 (18)	N3A—C2A—C10A	123.5 (2)
C3—C2—C10	119.79 (17)	C3A—C2A—C10A	121.05 (19)
C4—C3—C2	122.05 (18)	C4A—C3A—C2A	122.6 (2)
C8—C3—C2	119.78 (17)	C4A—C3A—C8A	118.0 (2)
C8—C3—C4	118.16 (18)	C8A—C3A—C2A	119.43 (19)
O1—C4—C3	124.19 (18)	O1A—C4A—C3A	123.2 (2)
O1—C4—C5	116.38 (18)	O1A—C4A—C5A	116.9 (2)
C5—C4—C3	119.43 (19)	C5A—C4A—C3A	119.8 (2)
C4—C5—H5	119.3	C4A—C5A—H5A	119.4
C6—C5—C4	121.38 (19)	C6A—C5A—C4A	121.3 (2)
C6—C5—H5	119.3	C6A—C5A—H5A	119.4
C5—C6—H6	120.0	C5A—C6A—H6A	120.1
C5—C6—C7	119.9 (2)	C5A—C6A—C7A	119.8 (2)
C7—C6—H6	120.0	C7A—C6A—H6A	120.1
O2—C7—C6	115.65 (18)	O2A—C7A—C6A	115.6 (2)
O2—C7—C8	124.61 (19)	C8A—C7A—O2A	124.3 (2)
C8—C7—C6	119.74 (19)	C8A—C7A—C6A	120.1 (2)
C3—C8—H8	119.3	C3A—C8A—H8A	119.5
C7—C8—C3	121.33 (19)	C7A—C8A—C3A	121.0 (2)
C7—C8—H8	119.3	C7A—C8A—H8A	119.5
O2—C9—H9A	109.5	O2A—C9A—H9AA	109.5
O2—C9—H9B	109.5	O2A—C9A—H9AB	109.5
O2—C9—H9C	109.5	O2A—C9A—H9AC	109.5
H9A—C9—H9B	109.5	H9AA—C9A—H9AB	109.5
H9A—C9—H9C	109.5	H9AA—C9A—H9AC	109.5
H9B—C9—H9C	109.5	H9AB—C9A—H9AC	109.5
C2—C10—H10A	109.5	C2A—C10A—H10D	109.5
C2—C10—H10B	109.5	C2A—C10A—H10E	109.5
C2—C10—H10C	109.5	C2A—C10A—H10F	109.5
H10A—C10—H10B	109.5	H10D—C10A—H10E	109.5
H10A—C10—H10C	109.5	H10D—C10A—H10F	109.5
H10B—C10—H10C	109.5	H10E—C10A—H10F	109.5
N1—C11—H11A	109.5	N1A—C11A—H11D	109.5
N1—C11—H11B	109.5	N1A—C11A—H11E	109.5
N1—C11—H11C	109.5	N1A—C11A—H11F	109.5
H11A—C11—H11B	109.5	H11D—C11A—H11E	109.5
H11A—C11—H11C	109.5	H11D—C11A—H11F	109.5
H11B—C11—H11C	109.5	H11E—C11A—H11F	109.5
N1—C12—H12A	109.5	N1A—C12A—H12D	109.5
N1—C12—H12B	109.5	N1A—C12A—H12E	109.5
N1—C12—H12C	109.5	N1A—C12A—H12F	109.5
H12A—C12—H12B	109.5	H12D—C12A—H12E	109.5
H12A—C12—H12C	109.5	H12D—C12A—H12F	109.5
H12B—C12—H12C	109.5	H12E—C12A—H12F	109.5
			
O1—C4—C5—C6	−178.0 (2)	O1A—C4A—C5A—C6A	−179.3 (2)
O2—C7—C8—C3	−179.22 (18)	O2A—C7A—C8A—C3A	−178.6 (2)
N2—N3—C2—C3	178.91 (16)	N2A—N3A—C2A—C3A	−177.70 (18)
N2—N3—C2—C10	−1.7 (3)	N2A—N3A—C2A—C10A	1.3 (3)
N3—N2—C1—S1	−2.4 (2)	N3A—N2A—C1A—S1A	−0.3 (3)
N3—N2—C1—N1	178.56 (17)	N3A—N2A—C1A—N1A	179.2 (2)
N3—C2—C3—C4	4.8 (3)	N3A—C2A—C3A—C4A	−2.1 (3)
N3—C2—C3—C8	−174.23 (17)	N3A—C2A—C3A—C8A	176.3 (2)
C1—N2—N3—C2	173.41 (17)	C1A—N2A—N3A—C2A	−171.6 (2)
C2—C3—C4—O1	−0.5 (3)	C2A—C3A—C4A—O1A	−2.7 (3)
C2—C3—C4—C5	179.65 (18)	C2A—C3A—C4A—C5A	177.1 (2)
C2—C3—C8—C7	178.66 (18)	C2A—C3A—C8A—C7A	−177.7 (2)
C3—C4—C5—C6	1.9 (3)	C3A—C4A—C5A—C6A	0.9 (4)
C4—C3—C8—C7	−0.4 (3)	C4A—C3A—C8A—C7A	0.7 (3)
C4—C5—C6—C7	−0.7 (3)	C4A—C5A—C6A—C7A	0.1 (4)
C5—C6—C7—O2	179.7 (2)	C5A—C6A—C7A—O2A	178.3 (2)
C5—C6—C7—C8	−1.0 (3)	C5A—C6A—C7A—C8A	−0.8 (4)
C6—C7—C8—C3	1.6 (3)	C6A—C7A—C8A—C3A	0.3 (3)
C8—C3—C4—O1	178.59 (18)	C8A—C3A—C4A—O1A	178.9 (2)
C8—C3—C4—C5	−1.3 (3)	C8A—C3A—C4A—C5A	−1.3 (3)
C9—O2—C7—C6	−176.3 (2)	C9A—O2A—C7A—C6A	−179.3 (2)
C9—O2—C7—C8	4.5 (3)	C9A—O2A—C7A—C8A	−0.3 (3)
C10—C2—C3—C4	−174.64 (18)	C10A—C2A—C3A—C4A	178.9 (2)
C10—C2—C3—C8	6.3 (3)	C10A—C2A—C3A—C8A	−2.8 (3)
C11—N1—C1—S1	−177.10 (15)	C11A—N1A—C1A—S1A	179.4 (2)
C11—N1—C1—N2	1.9 (3)	C11A—N1A—C1A—N2A	0.0 (3)
C12—N1—C1—S1	−0.7 (3)	C12A—N1A—C1A—S1A	3.7 (4)
C12—N1—C1—N2	178.29 (18)	C12A—N1A—C1A—N2A	−175.7 (2)

### Hydrogen-bond geometry (Å, º)

Cg1 and Cg2 are the centroids of the C3···C8 and C3A···C8A rings, respectively.

**Table:**

*D*—H···*A*	*D*—H	H···*A*	*D*···*A*	*D*—H···*A*
O1—H1···N3	0.84	1.84	2.563 (2)	143
O1*A*—H1*A*···N3*A*	0.84	1.86	2.565 (3)	141
C11—H11*A*···O1^i^	0.98	2.51	3.315 (3)	139
C11*A*—H11*E*···O1*A*^ii^	0.98	2.68	3.305 (4)	122
C11*A*—H11*E*···*Cg*2^iii^	0.98	2.73	3.590 (3)	147
C12—H12*B*···*Cg*1^i^	0.98	2.82	3.530 (3)	130

Symmetry codes: (i) −*x*+1, −*y*, −*z*+2; (ii) −*x*+1, −*y*, −*z*+1; (iii) −*x*+3/2, *y*+1/2, −*z*+1.
