# Supplementary figures and images for: The crystal structure of 1-(2-hydroxy-5-meth­oxy­phen­yl)ethanone 4,4-di­methyl­thio­semicarbazone
Source: Acta Crystallogr E Crystallogr Commun. 2015 Oct 3;71(Pt 11):o811–2. doi: 10.1107/S2056989015018228 (PMC4645029; doi:10.1107/S2056989015018228)

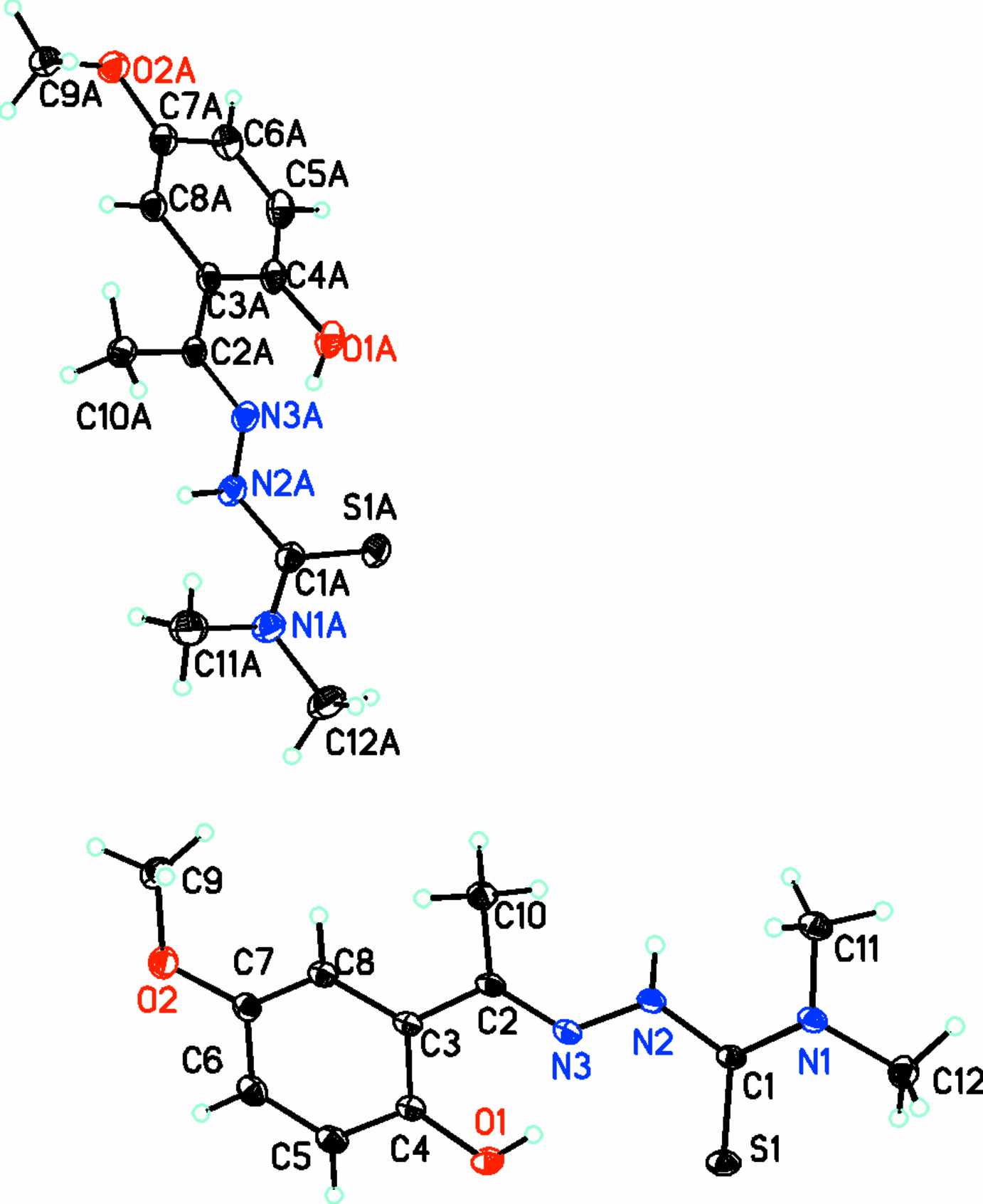

Supplement: Supplementary file 4 [file e-71-0o811-fig1.tif]

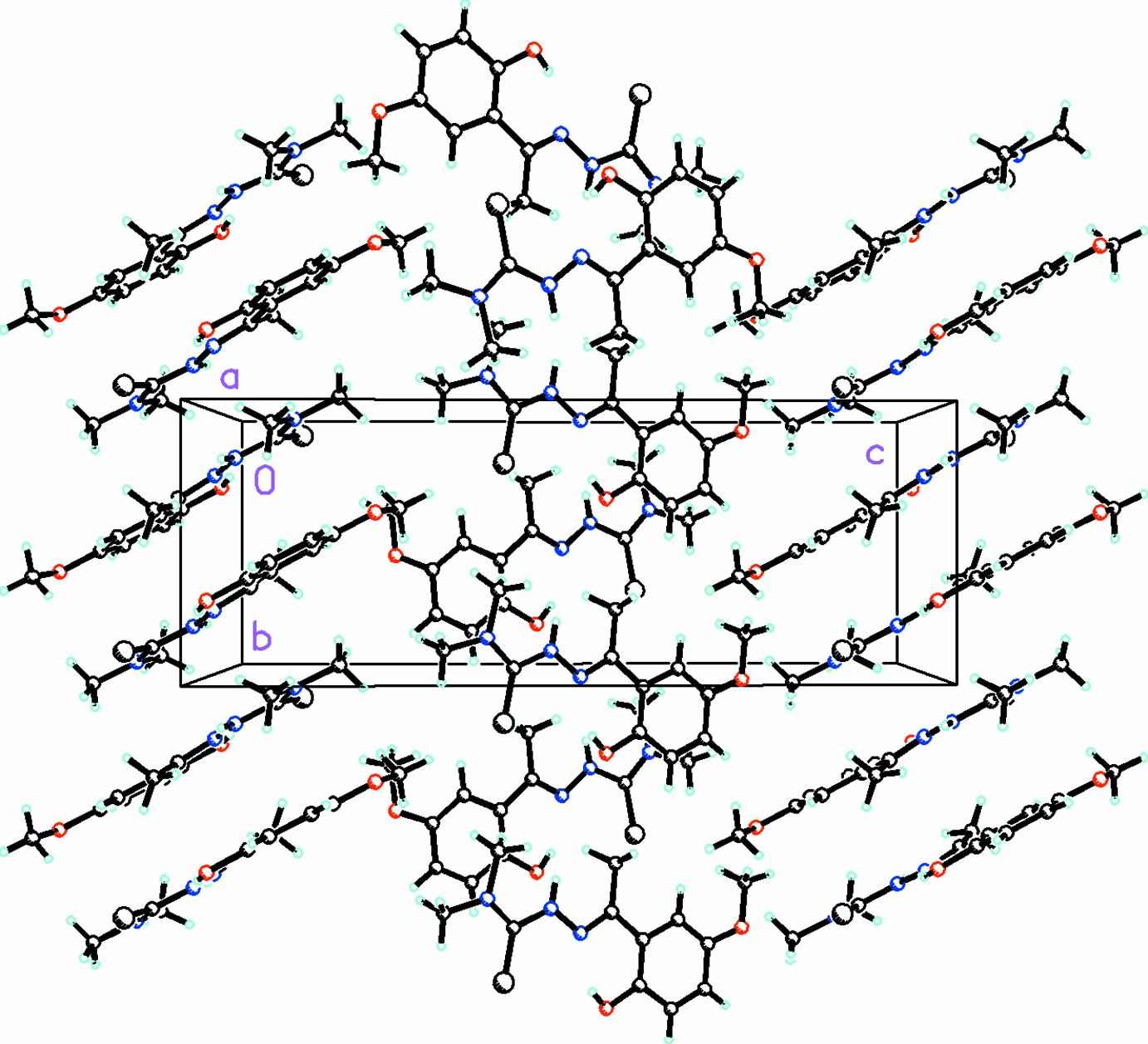

Supplement: Supplementary file 5 [file e-71-0o811-fig2.tif]
